# Dermal Substitutes and Skin Grafts in the Reconstruction of Post-Traumatic Total Scalp Avulsion: A Case Series

**DOI:** 10.3390/jcm12062167

**Published:** 2023-03-10

**Authors:** Marzia Petrocelli, Giulia Togo, Silvia Ricci, Flavia Zeneli, Sebastiano Cutrupi, Annamaria Baietti, Paola Bonavolontà, Luigi Califano, Luigi Angelo Vaira, Alfonso Scarpa, Arianna Di Stadio, Giovanni Salzano

**Affiliations:** 1 Maxillofacial Surgery Operative Unit, Bellaria and Maggiore Hospital, 47814 Bologna, Italy; annaria.baietti@ausl.bologna.it; 2 Maxillofacial Surgery Operative Unit, Department of Neurosciences, Reproductive and Odontostomatological Sciences, Federico II University of Naples, 80131 Naples, Italy; giulia.togo@gmail.com (G.T.); paola.bonavolonta@unina.it (P.B.); luigi.califano@unina.it (L.C.); giovannisalzanomd@gmail.com (G.S.); 3 Plastic Surgery Unit, Bellaria and Maggiore Hospital, 47814 Bologna, Italy; silvia.ricci01@ausl.bologna.it; 4 Department of Medical and Surgical Sciences, Division of Plastic Surgery, University of Modena and Reggio Emilia, Policlinico of Modena, Largo Pozzo 71, 41124 Modena, Italy; 297182@studenti.unimore.it; 5 Dentistry Operative Unit, Bellaria and Maggiore Hospital, 47814 Bologna, Italy; sebastiano.cutrupi@ausl.bologna.it; 6 Maxillofacial Surgery Operative Unit, Department of Medicine, Surgery and Pharmacy, University of Sassari, 07100 Sassari, Italy; luigi.vaira@gmail.com; 7 Biomedical Science Department, PhD School of Biomedical Science, University of Sassari, 07100 Sassari, Italy; 8 Department of Medicine and Surgery, University of Salerno, Via Salvador Allende 43, Baronissi, 84081 Salerno, Italy; alfonsoscarpa@yahoo.it; 9 Otolaryngology Department, University of Catania, 95124 Catania, Italy; ariannadistadio@hotmail.com

**Keywords:** scalp avulsion, scalp reconstruction, split-thickness skin graft (STSG), dermal substitutes

## Abstract

Although scalp defects can vary in size and thickness, scalp avulsion represents a rare occurrence. This type of lesion may have different origins, but it is usually related to long hair being caught in agricultural machinery. The management of full-thickness scalp defects poses a challenge to the head and neck surgeon due to the possible involvement of neurovascular structures and scar retraction, which can affect the esthetic restoration of the area. Several algorithms for the choice of scalp reconstruction have been proposed in the literature and different techniques are available for extensive scalp defect reconstruction (local soft tissue flap, microvascular free flap, and skin graft combined with dermal substitutes), based upon the scalp defect type. Here we describe six cases of patients with total scalp avulsion, which required a combined reconstruction with a split-thickness skin graft (STSG) and Integra^®^ matrix immediately after the trauma.

## 1. Introduction

Scalp avulsion is a rare occurrence, which may lead to permanent morbidity and psychological distress, often associated with pan-facial traumas [1]. Scalp defects can result from several different conditions, including traumatic lesions, burns, avulsion injuries, pressure sores, and primary malignancies.

Although many cases of scalp injury with variable size defects have been reported, complete scalp avulsion remains rare in clinical practice. The most common mechanism of injury is either hair entrapment in agricultural machinery in adults or animal bites in pediatric patients [2,3]. The traction forces on the hair commonly separate the galea aponeurotica from the periosteum, leaving the latter intact. However, the plane of cleavage may sometimes be irregular, presenting areas of damaged periosteum with exposure of the calvarium.

The management of patients with full-thickness scalp defects remains a challenge for the head and neck surgeon. The proper choice of reconstructive method is usually affected by the size, depth, and location of the defect, the injury to the periosteum, the quality of the surrounding scalp tissue, the damage to the hair, and any patient comorbidities.

The treatment of most scalp reconstructions must overcome the resistance of the tissue to distension, the potential involvement of neurovascular structures, and primarily the esthetic restoration of the area affected by the defect.

In extended scalp traumas, the primary goal is immediate reconstruction to prevent hemorrhage, desiccation, and infection. Secondary goals include the preservation of hair bearing skin and the hair contour, the restoration of quality tissues, and low scar contraction. Different techniques are available for extensive scalp defect reconstruction [4].

Based on the size of the scalp injury, the choice of flap for the reconstruction involves local flaps for small and medium full-thickness defects (2–25 cm^2^) [1], and free microvascular flaps or a skin regeneration model plus split-thickness skin grafts (STSG) for a full-thickness extensive reconstruction (>25 cm^2^) [2,3]. Skin grafts alone can only be obtained for partial-thickness defects in which pericranium has been spared.

The aim of this article is to present six cases of complete scalp avulsion, including partial damage to the periosteum, which required a combined reconstruction with an STSG and Integra^®^ matrix immediately after the trauma.

## 2. Materials and Methods

Six consecutive patients with scalp avulsion underwent a combined reconstruction with an STSG and Integra^®^ matrix between January 2020 and July 2022 at the Unità Operativa di Chirurgia del Volto Ausl Bologna Ospedale Bellaria e Maggiore di Bologna and the Unità Operativa di Chirurgia Plastica Ausl Romagna Ospedale M. Bufalini di Cesena.

The inclusion criteria were traumatic scalp avulsion with a follow-up period of at least 6 months after the surgery. The exclusion criteria consisted of patients with a concomitant cranial bone fracture and patients with a general contraindication to surgery.

All the patients were informed about the procedure and signed a pre-operative consent form for the recording of data in our clinical database. All the patients underwent a clinical facial analysis before the procedure and their medical histories were collected to identify risk factors, such as diabetes mellitus.

All six patients were admitted to the Emergency Department in the six hours after the trauma occurred. Upon admission CT scans were performed to exclude the presence of any associated life-threatening injuries. The amputated scalp delivered with the patient in a saline moistened gauze included all the hair-bearing scalp.

Integra^®^ is a dermal replacement, it is a synthetic skin replacement used to reconstruct wounds after elective scheduled surgery or after a trauma. It is based on a two-layer skin regeneration system. The outer layer consists of a thin silicone film that acts as the epidermis of the skin. The inner layer consists of a complex matrix of cross-linked fibers, which serves as a scaffold for the regeneration of skin cells, allowing the regrowth of a functional dermal layer of the skin.

### 2.1. Surgical Technique

**Figure 1:**
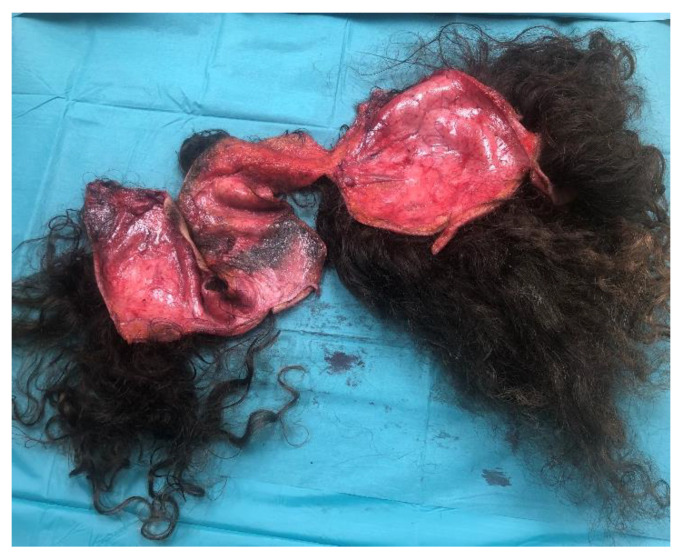
Avulsed scalp.

In each case, the amputated scalp delivered with the patient was observed through microscopic examination. No vessel was deemed adequate for replantation. In addition, the anatomical piece was highly contaminated (Figure 1).

**Figure 2:**
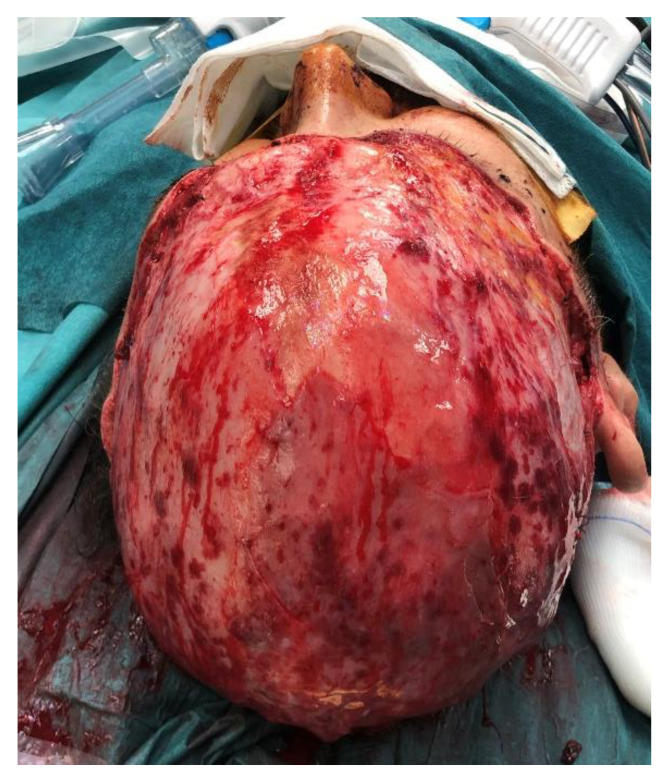
Wound surface after debridement.

Under general anesthesia, a pulsed lavage of the wound, meticulous debridement, and hemostasis were performed. Lacerated flaps of the scalp were repositioned in their anatomical sites. Any remaining parts of the galea were extended and sutured in order to leave most of wound surface covered by vascularized tissue (Figure 2).

**Figure 3:**
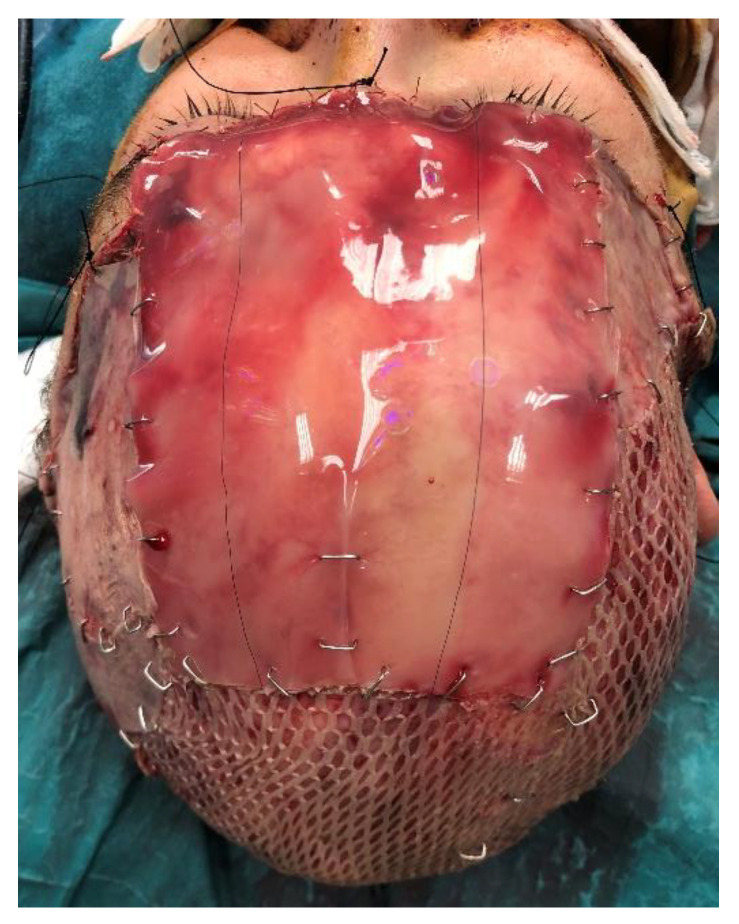
Positioning of the dermal substitute.

This allowed a reduction in the size of the exposed bone. In order to create a vascularized wound bed, the outer layer of the bone was debrided until bleeding from the diplopic space was visible and an Integra^®^ double layer was placed in these areas. STSGs from the patient’s thighs were meshed and applied to the rest of the wound. Care was taken to use a wide rectangle of Integra^®^ and unmeshed skin grafts in the frontal region to ensure a better esthetic outcome (Figure 3).

A tie-over bandage was placed all over the scalp.

At three weeks after the trauma a second surgery was performed to remove the silicone layer of Integra^®^ and to apply skin grafts where the neodermis was still present. The three-month post-surgery follow-up presented a completely healed wound, while the six-month post-surgery follow-up showed a satisfactory esthetic result.

### 2.2. Post-Surgical Assessment

All the patients underwent a regular post-operative follow-up at 1, 2, and 3 weeks as well as at 1, 3, and 6 months after the procedure.

Most of the grafts healed properly and the neodermis grew on the dermal matrix, except for two necrotic areas under 4 cm. These two minor necrotic areas were debrided, the exposed bone underneath was drilled to facilitate neodermis development, a new dermal matrix was applied and, three weeks later, coverage with an STSG was performed.

## 3. Results

Our sample consisted of six patients, four female and two male, with a mean age of 52.3 years (ranging from 39 to 67 years). Three of the patients were diabetic and one was diabetic with a history of heart disease. All the patients suffered from a traumatic scalp avulsion, with the mean defect size being 101 cm^2^ (ranging from 152 to 65 cm^2^). The avulsed areas involved are described in Table 1.

An STSG combined with an Integra^®^ matrix was performed immediately after the trauma. A second surgery was performed after three weeks to remove the silicone layer of the Integra^®^ and to apply skin grafts to the underlying neodermis. The average surgical time was between 60 min and 90 min depending on the lesion size. The mean hospitalization was 5 days, following which all patients were discharged in the absence of complications.

**Figure 4:**
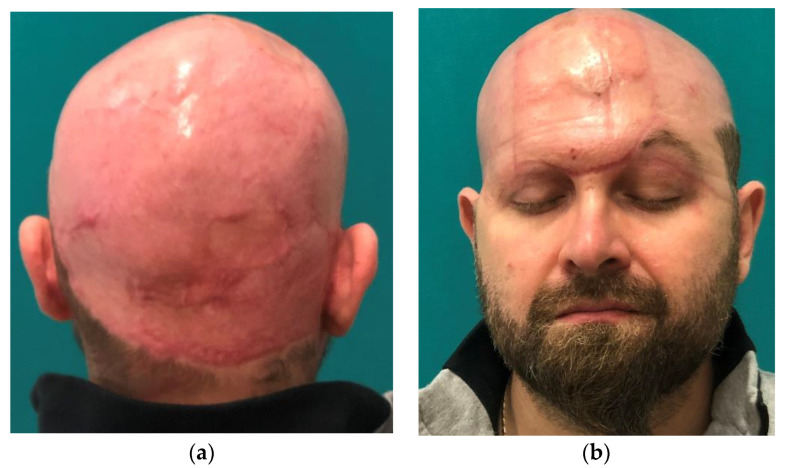
Post-operative images of one of the patients at three months. (**a**) posterior view (**b**) frontal view.

The three-month post-surgery follow-up showed a completely healed wound and no complications directly related to the procedure were reported. At the six-month post-surgery follow-up, no patients showed scar retractions that altered the morphology of the face, the retraction/hypertrophy of the surgical wound, or further complications. All the patients were satisfied with the esthetic and functional result of the procedure (Figure 4).

## 4. Discussion

Microsurgical techniques and dermal replacement technologies have advanced in the field of scalp reconstruction over the past decades. However, total scalp avulsion remains a challenge due to the large size of the defect. Moreover, ablative and traumatic defects of the head can also include the underlying bone, and therefore the pre-surgical planning should also evaluate the rigid reconstruction of any bone defects.

The main goals of any reconstruction of the scalp are the protection of the intracranial contents, while maintaining the contour of the head and providing an optimal esthetic restoration.

The scalp is the soft-tissue envelope of the caldaria vault. It includes five main tissue layers: the skin, connective tissue, the aponeurosis, loose areolar tissue, and the periosteum or pericranium (SCALP is the acronym commonly used as a mnemonic).

The scalp is vascularized by high-flow vessels, the superficial temporal and occipital arteries provide the main blood supply to this region.

In the literature there are several algorithms for the choice of scalp reconstructions, based on either the size, location, and etiology of the defect, or the quality of the tissue, the underlying structures exposure, and the distortion of the hairline.

Scalp defects are generally classified as small (up to about 10 cm^2^), medium (10–20 cm^2^), and large (over 20 cm^2^) [5,6,7].

Desai et al. [4] proposed an algorithm based on the size and location of the scalp defect, the history of radiation, and the hairline distortion.

Beasley et al. [8] proposed a staging system for scalp defects based exclusively on the position and size of the defect, considering possible reconstructions with local flaps for defects below 200 cm^2^.

Newman et al. considered the size of the defect and the quality of the tissue in choosing a surgical technique that allowed the use of local flaps even for defects larger than 50 cm^2^, but with the preliminary condition of good local tissue [6].

Secondary intention healing is a non-surgical option used for small defects in selected patients. The wound will contract by as much as 60% as it heals, potentially distorting the adjacent tissue, and it will close over in weeks to months, depending on its size and depth, through a process of granulation, contraction, and re-epithelialization.

Primary closure of scalp defects depends on the size and anatomical location. If the scalp defect is about 3 cm or less, it can usually be closed primarily. Based on the location of the defect, care must be taken to preserve the patient’s hairline without distorting the ipsilateral brow through excessive tension on the wound. As described by Sasaki et al. [9], cyclic expansions and deflations of a balloon device, such as a Foley catheter, beneath the closure site can assist in recruiting an additional 1 to 2 cm for the wound closure.

A local soft tissue flap or adjacent tissue transfer are usually used for small or medium sized defects, but may also be considered for the reconstruction of selected large defects [10]. Ibleher et al. [5] described a specific algorithm for oncological scalp reconstruction and considered a scalp defect from 6–8 cm or 4–5 cm at the hairline border after oncological surgery the threshold for using a local scalp flap. Local flaps can be categorized as advancement, rotation or transposition flaps, although some reconstructions are hybrids of these techniques. These flaps are considered relatively safe with a low major complication rate (3.4%) [6] and are considered an optimal surgical option in the treatment of patients who cannot undergo prolonged surgical procedures [11].

Microvascular free flaps have become the gold standard for the vascularized reconstruction of large (larger than 6–8 cm diameter) scalp defects where a local flap cannot provide reliable coverage [12]. Microvascular flap selection is highly variable and depends on the defect location, as well as the surgeon’s preference. While microsurgical replantation remains the main treatment, its feasibility depends on different factors, such as amputated scalp availability, ischemia time, and vessel viability. In both presented cases, vessel laceration and contamination of the scalp ruled out replantation. The disadvantage of this technique is the lengthening of the surgical and hospitalization times.

Skin grafting in the scalp area has a limited role due to suboptimal cosmesis and a lack of durability. The surgeon can select between an STSG or a full-thickness skin graft (FTSG) for the reconstruction, both grafts initially receiving nourishment by plasmatic imbibition. STSGs usually have better success rates than FTSGs due to the reduced metabolic requirements for thickness and the fact that they can generally cover a larger surface than an FTSG.

Schonauer et al. [13] described the use of STSGs to reconstruct major full-thickness scalp defects up to 350 cm^2^. A variety of dermal alternatives have been described for the coverage of scalp wounds. Integra^®^ (LifeScience Corp., Plainsboro, NJ, USA) and Matriderm (Dr Suwelack Skin and Health Care AG, Billerbeck, Germany) were first use in burn patients but their application was later expanded to reconstructive surgery in relation to chronic wounds [14,15]. Integra^®^ is a bilaminate synthetic construct consisting of an outer silicone layer and a porous inner collagen–glycosaminoglycan (chondroitin-6-sulfate) matrix. It is placed onto vascularized soft tissue, sutured into place, and secured with a bolster dressing for three to four weeks, so that the granulation tissue can prepare the wound bed to receive a staged skin graft. In some rare cases, it may be left to close secondarily.

To date, microsurgical replantation of the amputated scalp has been accepted as the gold standard treatment as it provides the best functional and esthetic result. Scalp replantation should also be considered, as multiple case series of avulsed scalp replantation have shown significant success rates [16,17].

Taking into account the fact that the most common etiology of scalp avulsion is hair entrapment in mechanical devices for agriculture purposes [2], the microsurgical replantation of the scalp may not be possible due to patient comorbidity, the absence of vessels suitable for anastomosis, and the contamination of the anatomical piece by oily substances and small metal fragments from the machinery.

However, when this is unachievable or unsuccessful, other reconstructive options such as skin grafts, often combined with dermal substitutes, represent an adequate option in the reconstruction of full-thickness scalp defects. According to the literature [13], the combination of an STSG and a dermal substitute provides an excellent esthetic restoration and a low rate of scarring complications.

Our case history includes six patients, who had suffered a traumatic total scalp avulsion following an agricultural machinery accident. The microsurgical replantation of the avulsed scalp was not performed due to patient comorbidity and the lack of adequate vessels. All the patients were treated with an STSG combined with a dermal substitute (Integra^®^). In each case, the post-operative follow-up showed a satisfactory restoration of the facial esthetics, in the absence of pathological scar retractions and complications related to the surgical procedure.

## 5. Conclusions

In conclusion, scalp reconstruction is a complex field with numerous surgical options and different approaches available. The successful reconstruction of the scalp requires an accurate and individualized approach with attention to both the functional and esthetic goals.

An immediate evaluation should be performed to assess whether replantation is possible due to the increased risk of infection in the case of massive necrosis of the scalp. When this is not possible, reconstruction with a skin graft, in an emergency setting, is generally feasible with satisfactory functional and esthetic results.

In the literature there is no long-term retrospective study with a large sample size and no evidence of guidelines for repairing emergency scalp avulsions or defects in critical conditions. In our experience, the use of dermal matrixes in the area of an exposed skull bone has enabled a total coverage in an emergency setting, reducing the risk of infection and shortening the recovery time. This procedure has provided a satisfactory esthetic restoration, without any aberrant scarring tissue, and with a low risk of post-operative complications.

## Figures and Tables

**Table 1:** General characteristics of the patients.

Case	Age	Sex	Mechanism	Dimension (cm^2^)	Anatomical Area
1	41	F	Agricultural accident	152	All the hair-bearing scalp until the nuchal area, the forehead skin, the entire right eyebrow, part of the right eyelid and part of the left eyebrow
2	67	M	Agricultural accident	136	All the hair-bearing scalp until the nuchal area, the forehead skin, the entire right eyebrow, part of the right eyelid and part of the left eyebrow
3	39	F	Agricultural accident	65	Part of the hair-bearing scalp of the forehead skin
4	59	F	Agricultural accident	80	All the hair-bearing scalp until the nuchal area, with the forehead skin above the eyebrows not involved
5	57	F	Agricultural accident	73	The hair-bearing scalp of the forehead skin and nasal area
6	51	M	Agricultural accident	100	All the hair-bearing scalp until the nuchal area, with the forehead skin above the eyebrows not involved

## Data Availability

The data presented in this study are available on request from the corresponding author. The data are not publicly available due to reasons of privacy.
